# The Evolving Molecular Landscape of Uterine Mesenchymal Tumors: Diagnostic and Therapeutic Implications

**DOI:** 10.3390/cancers17244012

**Published:** 2025-12-16

**Authors:** Tong Sun

**Affiliations:** Department of Pathology, Yale School of Medicine, Yale University, New Haven, CT 06520-8023, USA; tong.sun@yale.edu

**Keywords:** uterine mesenchymal tumors, molecular genetics, precision oncology

## Abstract

Uterine mesenchymal tumors encompass a diverse and diagnostically challenging group of neoplasms, including smooth muscle tumors, endometrial stromal tumors (ESS), perivascular epithelioid cell tumors (PEComas), inflammatory myofibroblastic tumors (IMTs), uterine tumor resembling ovarian sex cord tumor (UTROSCT), along with many other relatively rare entities. Traditionally classified by histomorphology and immunophenotype, these tumors are now increasingly defined by recurrent genetic alterations that refine diagnosis and elucidate tumorigenesis. For example, leiomyosarcomas display complex genomic instability with frequent *TP53*, *RB1*, and *ATRX* mutations. Low grade-ESS are characterized by *JAZF1*::*SUZ12* and other related fusions, whereas high-grade tumors harbor *YWHAE*::*NUTM2* or *ZC3H7B::BCOR* fusions, and *BCOR* internal tandem duplication (ITD) alterations. PEComas frequently contain *TSC1* or *TSC2* mutations, leading to aberrant activation of the mTOR pathway. Beyond their diagnostic utility, these molecular signatures increasingly inform prognosis and highlight potential therapeutic targets, including CDK4/6 inhibition, PI3K/AKT/mTOR blockade, and immunotherapy. This review summarizes the evolving molecular landscape of uterine mesenchymal tumors, underscoring the value of integrating molecular testing into clinical practice to enhance diagnostic precision and enable personalized management of these rare yet clinically significant neoplasms.

## Simple Summary

Uterine mesenchymal tumors are rare cancers that are difficult to classify using morphology alone. This review highlights how recurrent genetic alterations now define key tumor subtypes, improve diagnostic accuracy, and reveal actionable therapeutic targets. Integrating molecular testing into routine practice enables more precise diagnosis and supports personalized treatment, with the potential to improve patient outcomes.

## 1. Introduction

Uterine mesenchymal tumors encompass a morphologically and molecularly heterogeneous spectrum of neoplasms derived from smooth muscle, endometrial stroma, or uncertain origins [1]. Despite the rarity of most entities, they pose considerable diagnostic and management challenges owing to overlapping histologic features and unpredictable biological behavior [2,3].

Traditional classification based on morphology and immunophenotype has been revolutionized by the advent of high-throughput genomic and transcriptomic profiling, which has revealed recurrent, tumor-type-specific genetic alterations. These discoveries have redefined disease taxonomy, improved diagnostic accuracy, and provided new insights into tumor biology and therapeutic vulnerability [3,4].

This review summarizes the molecular and genetic foundations of the major categories of uterine mesenchymal tumors, including smooth muscle tumors, endometrial stromal tumors, PEComas, IMT, UTROSCT and other emerging molecularly defined sarcomas, and discusses their implications for diagnosis, classification, and clinical management.

## 2. Uterine Smooth Muscle Tumors

Uterine smooth muscle tumors represent a diverse group of neoplasms that range from benign leiomyomas to highly aggressive leiomyosarcomas [5]. Although they share morphologic features derived from smooth muscle differentiation, their biological behavior and molecular underpinnings differ markedly [6]. Extensive genomic and transcriptomic studies have demonstrated that leiomyomas and leiomyosarcomas rarely share common driver mutations, indicating that these tumors arise through distinct molecular pathways rather than representing points along a single neoplastic continuum [7]. Understanding these divergent mechanisms is essential for improving diagnostic accuracy, prognostication, and the development of targeted therapeutic strategies.

### 2.1. Uterine Leiomyomas

Uterine leiomyomas, commonly known as fibroids or uterine myomas, represent the most prevalent benign neoplasm of the female reproductive tract, affecting up to 70% of women during their lifetime [8]. Clinically, they are the leading indication for hysterectomy and a major cause of infertility and abnormal uterine bleeding. Consequently, uterine leiomyomas impose a substantial burden on women’s quality of life and contribute significantly to global healthcare costs [8,9].

The pathogenesis of uterine leiomyoma involves a complex interplay of germline susceptibility factors and somatic genetic alterations, including point mutations and chromosomal abnormalities. These molecular events both initiate and sustain tumor development [9]. Cytogenetic analyses have revealed recurrent structural rearrangements affecting several chromosomes, most frequently 7, 12, 14, and 15 [9,10,11]. Among these, the most frequent somatic mutation occurs in the *MED12* gene, which is altered in up to 70% of leiomyomas (Table 1) [11,12]. In uterine leiomyomas, *MED12* gene exon 2 mutations impair the normal function of the mediator complex, a key transcriptional regulator, leading to dysregulated transcriptome that promotes tumor growth (“gain of function”). These mutations disrupt the activation of Cyclin C-dependent kinases CDK8 and CDK19, resulting in altered transcription of multiple downstream targets within key signaling pathways including Wnt/*β*-catenin, hedgehog, and TGF-*β*. These changes ultimately drive the abnormal proliferation and development of leiomyoma cells [12,13].

Other tumors show chromothripsis-like rearrangements involving limited chromosomal regions, a catastrophic event in which chromosomes undergo massive rearrangements in one or a few mitotic cycles, observed in approximately 20–40% of cases [11,14,15].

Rearrangements at 12q14-15 lead to overexpression of *HMGA2*, a non-histone chromatin-binding protein implicated in transcriptional regulation [9,10,16]. Aberrant HMGA2 expression contributes to leiomyoma development through multiple mechanisms, including enhanced angiogenesis, estrogen receptor (ER)-mediated proliferation, and impaired homologous recombination repair via translocation with the *RAD51B* gene [17]. Similarly, *HMGA1*, located at 6p21, can be affected by chromosomal rearrangements, and disruptions of *HMGA1* or *HMGA2* result in comparable downstream biological effects. In addition, deletions involving the X chromosome that affect *COL4A5* and *COL4A6*, genes associated with Alport syndrome, have been linked to diffuse leiomyomatosis and uterine leiomyoma formation [18].

Epigenetic dysregulation also plays an important role in uterine leiomyomas pathogenesis. Compared to normal myometrium, leiomyomas exhibit aberrant DNA methylation patterns, increased expression of ER, and elevated levels of DNA methyltransferases [19]. Upregulation of HMGA2 may also occur via hypomethylation rather than chromosomal translocation [20]. Moreover, DNA methylation changes interact with *MED12* mutations to form a regulatory network that modulates progesterone receptor (PR) mediated RANKL expression, promoting stem cell proliferation and tumor growth [19,20,21]. Collectively, these findings highlight the central role of genetic and epigenetic alterations in the molecular pathogenesis of uterine leiomyomas and underscore potential avenues for targeted therapeutic intervention.

FH-deficient uterine leiomyomas represent a distinct variant occurring sporadically or in the setting of hereditary leiomyomatosis and renal cell carcinoma (HLRCC), caused by inactivating *FH* mutations that lead to loss of enzyme activity and fumarate accumulation [22,23,24]. Excess fumarate functions as an oncometabolite, driving protein succination through accumulation of S-(2-succinyl) cysteine (2SC), a stable post-translational modification formed by nonenzymatic reaction of fumarate with cysteine residues, along with Hypoxia-Inducible Factor (HIF)-mediated pseudohypoxia, and global epigenetic dysregulation, collectively promoting tumor initiation and progression [22,23]. Morphologically, these tumors show macronucleoli with perinucleolar halos, staghorn vasculature, and eosinophilic globules, and they characteristically demonstrate loss of FH expression with strong 2SC positivity [24,25,26]. In recent cohorts, 26–34% of genetically tested patients with FH-deficient uML harbored germline *FH* variants [24,25,26]. Recognition of these features is essential for identifying patients at risk for HLRCC and guiding appropriate genetic evaluation and renal surveillance (Table 1).

### 2.2. Intravenous Leiomyomatosis (IVL)

IVL is a rare benign smooth muscle tumor characterized by its origin in the uterus and its potential intrapelvic or extra-pelvic extension along the venous system. Although histologically benign, IVL demonstrates a distinctive growth pattern that may confer a low-grade malignant potential [27].

The pathogenesis of IVL remains incompletely understood, but molecular studies have provided insights into its genetic background. Cytogenetic analyses have shown that IVL shares chromosomal alterations with typical uterine leiomyomas, including rearrangements involving 12q14–15 that affect the *HMGA2* gene, which plays a role in stem cell self-renewal [28,29]. Additional chromosomal abnormalities have been reported at 1p, 22q, 2q, 1q, 13q, 3q, and 10q, involving genes implicated in mesenchymal tumorigenesis and supporting the concept that IVL represents a neoplasm with intermediate, quasi-malignant biological behavior (Table 1) [28,29,30].

Unlike uterine leiomyomas, which frequently harbor *MED12* exon 2 mutations, most IVLs display wild-type *MED12* or distinct variants, suggesting a different molecular pathogenesis [28,29,30]. Transcriptomic profiling has demonstrated both overlapping and distinct expression signatures between IVL and uterine leiomyoma, with IVL showing elevated *HOXA13* expression, upregulation of anti-apoptotic genes such as *BCL2A1* and *CDKN2A*, and downregulation of the angiogenesis-related gene *CXCL8*. These findings highlight the molecular distinctiveness of IVL and provide potential biomarkers for differentiating it from uterine leiomyoma, while also offering insights into its unique biological behavior [28,29,30,31].

### 2.3. Smooth Muscle Tumors of Uncertain Malignant Potential (STUMPs)

STUMPs account for approximately 2–5% of all uterine smooth muscle neoplasms and pose significant diagnostic and management challenges due to their unpredictable biological behavior [32,33]. By definition, STUMPs exhibit at least one of the three histologic criteria used for diagnosing leiomyosarcoma, including coagulative tumor cell necrosis, cytologic atypia, or elevated mitotic activity, but not enough features to warrant a definitive diagnosis of malignancy. Additional histopathologic clues that may suggest aggressive potential include atypical mitoses, vascular invasion, and infiltrative or irregular tumor margins. According to the 2020 WHO classification, a STUMP is defined as “a smooth muscle tumor with features that preclude an unequivocal diagnosis of leiomyosarcoma, but do not fulfill the criteria for leiomyoma or its variants and raise concern that the neoplasm may behave in a malignant fashion” [1,32,33]. The advent of molecular profiling has provided promise in refining the prognostic assessment of these tumors.

Comparative molecular studies have shown that atypical leiomyomas and leiomyosarcoma share similar microRNA signatures. In one analysis referencing both the WHO and Stanford classifications, atypical leiomyoma, STUMP, and leiomyosarcoma demonstrated significantly higher frequencies of *TP53* mutations and *PTEN* deletions than conventional leiomyoma and leiomyoma variants. These findings suggest a partial molecular overlap among atypical leiomyoma, STUMP, and leiomyosarcoma [34]. However, despite their shared molecular alterations, most atypical leiomyoma and STUMP cases exhibit histologic and clinical features that are distinctly less aggressive than those of leiomyosarcoma [32,33,34].

Additional molecular markers have also been explored in STUMP. Loss of *ATRX* and *DAXX* expression, often resulting from underlying mutations, has been correlated with poor prognosis [35]. Using array-comparative genomic hybridization (array-CGH), Croce et al. developed a genomic profiling approach for uterine smooth muscle tumors. Among 14 STUMP cases, those with a genomic index (GI) < 10 had no recurrence, whereas 7 of 12 with GI ≥ 10 experienced relapse [36]. This study group proposed that genomic profiling could stratify STUMPs into two categories: a benign-like group resembling leiomyoma and a potentially malignant group with recurrence risk [36,37]. They advocated that tumors with GI > 10 undergo additional testing using the Complexity Index in SARComa (CINSARC) transcriptomic signature, a surrogate marker for chromosomal complexity and instability [38]. The CINSARC signature may not only identify patients at increased risk of recurrence or death but also provide insight into metastatic pathways and potential therapeutic targets. Tumors with a higher burden of copy number alterations were associated with worse clinical outcomes [38].

Several unusual smooth muscle tumor variants, including IVL, benign metastasizing leiomyoma, leiomyoma with bizarre nuclei, and diffuse leiomyomatosis, can exhibit worrisome morphologic features overlapping with STUMP, occasionally leading to diagnostic ambiguity [33,34]. With the expanded use of molecular techniques, it has become evident that some tumors previously classified as STUMP belong to distinct non-smooth muscle neoplasm categories, such as high-grade endometrial stromal sarcoma (HG-ESS) with *BCOR* alterations, uterine sarcomas with *NTRK* rearrangements or *COL1A::PDGFRB* fusions, and IMTs [3]. These entities can mimic smooth muscle tumors both morphologically and immunohistochemically, suggesting that the historical STUMP category may have encompassed a heterogeneous group of lesions that are now being reclassified based on their molecular signatures.

### 2.4. Uterine Leiomyosarcoma

Uterine leiomyosarcoma is a rare and aggressive mesenchymal neoplasm arising from the myometrium, accounting for approximately 2–5% of all uterine malignancies [39]. Despite its low incidence, uterine leiomyosarcoma represents a major clinical challenge due to its unpredictable biologic behavior, high recurrence rate, and poor overall prognosis. Overall, the 5-year survival rate for uterine leiomyosarcoma is approximately 25–76%, with much poorer prognosis for those with metastatic disease at the time of diagnosis, where rates can drop to 10–15% [40,41]. Accurate diagnosis remains difficult, particularly in distinguishing uterine leiomyosarcoma from other smooth muscle tumors such as atypical leiomyoma and STUMP, given overlapping morphologic and immunophenotypic features [34,39,40].

Over the past decade, increasing use of genomic and transcriptomic profiling has provided valuable insights into the molecular pathogenesis of uterine leiomyosarcoma and opened potential avenues for diagnostic refinement [42,43]. However, the genetic underpinnings of uterine leiomyosarcoma are complex, reflecting extensive genomic instability and a high degree of intertumoral heterogeneity. Cytogenetic analyses have revealed that uterine leiomyosarcoma typically exhibits highly aberrant karyotypes, frequently showing aneuploidy and polyploidy. Large-scale genomic alterations are common, including copy number gains affecting up to 15% and deletions involving up to 45% of the genome [44]. The uterine leiomyosarcoma genome also frequently demonstrates chromothripsis, resulting in concurrent loss of tumor suppressor genes and activation of oncogenic drivers [44,45]. These molecular hallmarks highlight the profound genomic instability that drives tumor initiation and progression in uterine leiomyosarcoma.

Recent molecular studies have identified two clinically relevant, pan-cancer biomarkers of DNA damage repair deficiency in subsets of uterine leiomyosarcoma: homologous recombination deficiency (HRD) and mismatch repair (MMR) deficiency, the latter leading to microsatellite instability (MSI) [45,46]. HRD-positive tumors may show sensitivity to poly (ADP-ribose) polymerase (PARP) inhibitors, while MMR-deficient leiomyosarcoma may respond to immune checkpoint blockade therapies (Table 2) [46]. However, these molecular alterations appear to occur in a minority of cases, and their predictive and prognostic value remains uncertain. Importantly, neither HRD nor MSI testing has yet been incorporated into the standard clinical management guidelines for uterine leiomyosarcoma [45,46]. Characterization of these subgroups will be critical to identify patients who may benefit from emerging precision medicine strategies (Table 2).

The genetic landscape of uterine leiomyosarcoma is primarily defined by recurrent alterations in genes regulating the cell cycle, apoptosis, and DNA repair, as well as by activation of alternative telomere lengthening (ALT) mechanisms [40,41,42,43,44,45]. The most frequently affected pathways include *TP53*, *RB1*, and *PTEN*, which play central roles in tumor suppression and genomic integrity. Loss of RB1 function, through either deletion or inactivation, disrupts cell cycle control and promotes uncontrolled cellular proliferation. Concurrently, *TP53* mutations lead to evasion of apoptosis and enhanced genomic instability. Alterations in *PTEN*, a negative regulator of the PI3K/AKT/mTOR signaling pathway, further contribute to tumor growth and resistance to apoptosis [39,40,41,42,43,44,45].

A characteristic feature of uterine leiomyosarcoma is the frequent involvement of the *ATRX* and *DAXX* genes, which regulate chromatin remodeling and telomere maintenance. Loss-of-function mutations in these genes lead to activation of the ALT pathway, a telomerase-independent mechanism that sustains telomere length and contributes to the immortalization of tumor cells [45,47,48]. A poor genomic index associated with *ATRX* and *DAXX* mutations is linked to increased genomic instability and poor prognosis in several cancers, including pancreatic neuroendocrine tumors (PanNETs) and glioma [49]. Immunohistochemical (IHC) loss of ATRX or DAXX protein expression is now recognized as a useful surrogate marker for these alterations and can aid in the classification of uterine smooth muscle tumors with ambiguous histologic features [48]. Other IHC markers such as p16, p21, Ki-67, stathmin-1, BCL2, and PHH3 have also been evaluated for their diagnostic and prognostic value, reflecting the molecular complexity of these tumors (Table 1) [50].

In a recent comprehensive study, investigators characterized the genomic and proteomic profiles of uLMS and identified surrogate IHC markers that could support molecular classification and diagnostic evaluation of challenging cases. Using next-generation sequencing (NGS), the study revealed frequent *RB1* deletions, *TP53* mutations, and alterations in other key regulators of the cell cycle, including *CDKN2A*, *CDKN2C*, and *MDM2*, as well as *PTEN* deletions and *ATRX*/*DAXX* mutations [45,47]. These genetic alterations exhibited mutual exclusivity within their respective pathways, for example, between *TP53*, *MDM2*, and *CDKN2A*, or between *ATRX* and *DAXX*, indicating alternative but functionally convergent routes of tumorigenesis. Corresponding protein expression abnormalities were confirmed by IHC, validating the use of these markers as practical surrogates for molecular testing (Table 1). Based on these findings, the authors proposed an integrated molecular-IHC diagnostic algorithm for the classification of uterine smooth muscle tumors that do not fulfill traditional morphologic criteria for malignancy [47].

Rare variants of uterine leiomyosarcoma include epithelioid and myxoid types, which can be histologically distinct from the common spindle cell form [39,51]. *PGR* gene fusions define a subset of uterine epithelioid leiomyosarcoma, with recurrent rearrangements such as *PGR::NR4A3* representing the most commonly reported events [51,52]. The fusion typically involves exon 2 of the *PGR* gene joining with exon 2 (or the entire coding sequence) of the *NR4A3* gene. This results in a hybrid protein containing the progesterone receptor domain of *PGR* and the full *NR4A3* protein sequence. it leads to an aberrant, increased transcriptional activation of *NR4A3* target genes compared to the wild-type *NR4A3* protein. This novel activity promotes cell proliferation and inhibits differentiation. These molecular alterations are associated with a distinctive biphasic morphology, observed in up to 35% of cases, in which epithelioid round and spindle-shaped tumor cells coexist. This biphasic growth pattern helps identify this specific molecular subtype and distinguish it from conventional leiomyosarcoma or other spindle cell neoplasms of the uterus [51,53,54]. Histologically, the spindle cell component is often low-grade, although occasional high-grade areas may be present. Immunohistochemically, these tumors are typically positive for desmin, ER, and PR, while they generally lack expression of CD10 and HMB45 [51,53]. Clinically, *PGR* fusion uterine leiomyosarcomas are considered an aggressive subset, with a potential for local recurrence and, in some cases, distant metastasis [51,53,54]. The recognition of this fusion is important not only for accurate diagnosis but also for prognostic stratification and potential consideration in the context of targeted therapy or clinical trials, given the gene-specific molecular alteration.

**Figure 1:**
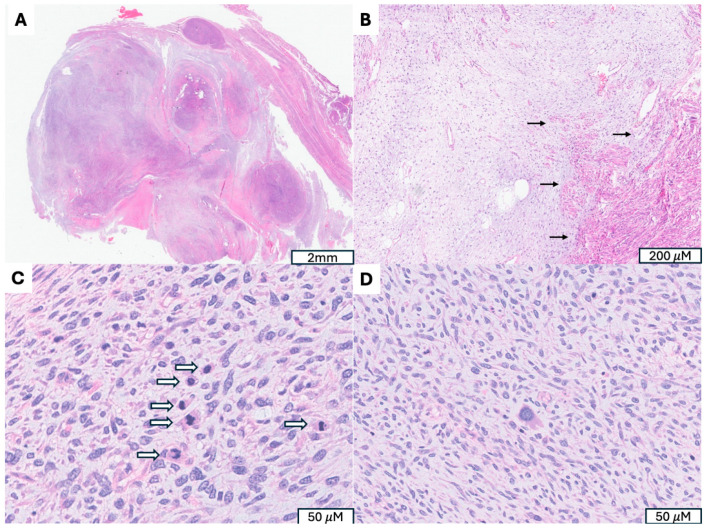
Uterine Myxoid Leiomyosarcoma Associated with *PLAG1* Gene Rearrangement. (**A**) A 12 cm tumor with nodular growth pattern and abundant myxoid stroma. (**B**) Hypocelluar myxoid stroma with an infiltrative border (indicated by →). (**C**) Increased mitotic activity (mitotic figures indicated by ⇒) (**D**) Moderate to severe cytologic atypia, including hyperchromatic and pleomorphic nuclei, irregular nuclear membranes present.

*PLAG1* gene fusions are identified in approximately 25% of myxoid leiomyosarcomas, a distinct subtype of soft tissue sarcoma (Figure 1) [55,56]. The *PLAG1* gene encodes a zinc finger transcription factor that regulates cell growth and proliferation. It is recurrently rearranged in several tumor types, most notably pleomorphic adenomas of the salivary glands. Oncogenic activation typically occurs through gene fusions or chromosomal translocations that relocate the *PLAG1* coding region under the control of strong heterologous promoters or enhancers, leading to its aberrant overexpression [55,56]. PLAG1 protein overexpression serves as a useful diagnostic biomarker for recognizing this specific tumor type [55]. The fusion arises from a chromosomal rearrangement that places *PLAG1* under the regulatory control of the partner gene’s promoter, leading to constitutive activation and increased protein expression. While the specific partner gene can vary, the pathogenic consequence remains similar [55,56,57]. Detection of *PLAG1* fusions, using fluorescence in situ hybridization (FISH) or PLAG1 IHC, may aid in distinguishing myxoid leiomyosarcoma from other histologically similar tumors (Table 1). Intriguingly, *PLAG1* gene fusions are also observed in a spectrum of other benign and malignant neoplasms with variable morphology, including pleomorphic adenomas, lipoblastomas and pediatric fibromyxoid soft tissue tumors [52,55,56].

These studies underscore the marked heterogeneity and genomic instability of uterine leiomyosarcoma, highlighting recurrent alterations in cell-cycle regulation, DNA repair, and telomere maintenance. While clinical translation remains limited, these insights are progressively improving diagnostic precision and informing more individualized therapeutic strategies.

## 3. Endometrial Stromal Tumors

Endometrial stromal tumors are rare and biologically fascinating uterine mesenchymal neoplasms characterized by diverse histologic, immunophenotypic, and molecular features. Morphologically, they recapitulate the proliferative-phase endometrial stroma [58]. Historically, Norris and Taylor (1966) classified these tumors into benign and malignant categories based primarily on mitotic activity [59]. However, molecular discoveries over the past decades have revealed a broader biological spectrum. In the 2020 WHO classification, endometrial stromal tumors are divided into four distinct categories, including endometrial stromal nodule (ESN), low-grade endometrial stromal sarcoma (LG-ESS), HG-ESS, and undifferentiated uterine sarcoma (UUS), reflecting their unique morphologic and genetic profiles [1].

### 3.1. Endometrial Stromal Nodule (ESN)

ESNs are benign, well-circumscribed proliferations composed of uniform spindle to oval cells with scant cytoplasm, resembling proliferative-phase endometrial stromal cells. Numerous small arterioles are typically present. The defining feature is their expansile, non-infiltrative margins; however, minimal irregularity or limited infiltration (<3 mm in depth or ≤3 foci) may occasionally occur. Exceeding these limits precludes a diagnosis of ESN [58,60].

The key distinction between ESN and LG-ESS lies in the growth pattern. ESNs exhibit smooth, pushing borders and lack lymphovascular invasion (LVI), whereas LG-ESS demonstrates infiltrative, tongue-like extensions into the myometrium and/or vascular spaces. Therefore, a definitive diagnosis of ESN requires evaluation of the entire tumor margin, which cannot be reliably assessed on limited specimens such as endometrial curettings, biopsies, or tissue removal system samples (e.g., MyoSure resections). Even in resection specimens, extensive sampling is essential to exclude focal infiltration characteristic of LG-ESS [60,61].

Molecularly, some ESNs harbor fusions also found in LG-ESS, particularly *JAZF1::SUZ12* or *JAZF1::PHF1*. Rare fusions such as *MEAF6::PHF1* have also been identified in ESNs, supporting the concept of a molecular continuum within the endometrial stromal tumor family. The large prospective studies are needed for tumor harboring such gene fusions to determine their prognostic significance [60,61,62,63].

Beyond fusion profiling, emerging studies suggest that epigenetic and DNA methylation analyses may offer additional diagnostic precision, with distinct methylation signatures at specific loci serving as promising biomarkers to distinguish benign from malignant stromal tumors and to improve reproducibility in borderline cases [61,62].

### 3.2. Low-Grade Endometrial Stromal Sarcoma (LG-ESS)

**Figure 2:**
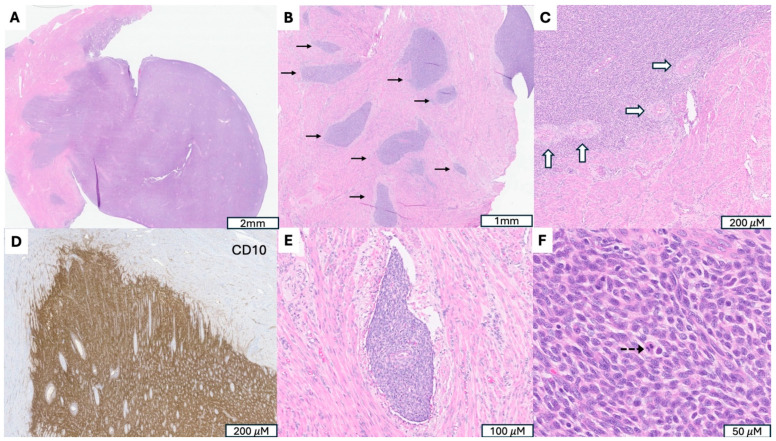
A Low-Grade Endometrial Stromal Sarcoma (LG-ESS) with *JAZF1::SUZ12* Fusion. (**A**) Tumor arising in endometrium and presenting as a polypoid mass. (**B**) Irregular cellular islands (indicated by →) forming tongue-like pattern myometrial invasion. (**C**) Perivascular whirling of tumor cells around arteriolar type vessels (indicated by ⇒), reminiscent of proliferative endometrial stroma. (**D**) Strong CD 10 immunoreactivity in tumor cells, supporting endometrial stromal origin. (**E**) Parametrial vascular invasion, a hallmark feature indicating malignant potential and worse prognosis. (**F**) Monotonous oval to spindle tumor cells with minimal cytologic atypia, vesicular chromatin, scant cytoplasm and occasional mitotic figures (indicated by -->).

LG-ESS closely resembles ESN histologically but demonstrates infiltrative growth and LVI, often extending into parametrial vessels. Microscopically, it features small, uniform stromal cells forming permeative, tongue-like projections into the surrounding myometrium. Mitotic activity is generally low, and necrosis is uncommon (Figure 2). Immunohistochemically, LG-ESS is typically negative for Cyclin D1 and BCOR, markers that are frequently expressed in HG-ESS [60,61,63,64,65].

Molecularly, LG-ESS is genetically heterogeneous, driven by a range of recurrent chromosomal translocations. Approximately one-third of cases lack detectable gene fusions. The *JAZF1::SUZ12* fusion is the most frequent, present in roughly half of LG-ESS and some ESNs, and is considered a cytogenetic hallmark of this group [63,64]. *JAZF1* encodes a zinc-finger transcriptional repressor. SUZ12 is a core component of the polycomb repressive complex 2 (PRC2) and mediates chromatin compaction and gene silencing, while PHF1 is an accessory protein that interacts with PRC2 and PRC1 to help target polycomb complexes to chromatin. Gene fusions involving *JAZF1::SUZ12* or *JAZF1::PHF1* produce chimeric proteins that attenuate PRC repressive function and alter histone methylation patterns, particularly by reducing H3K27me3, thereby creating a more permissive environment for cell proliferation [65]. Additional reported fusions include those involving *PHF1* (*EPC1::PHF1*, *MEAF6::PHF1*, *BRD8::PHF1*, *EPC2::PHF1*), *JAZF1* (*JAZF1::BCORL1*, *SYNGAP1::JAZF1*), and other chromatin-remodeling genes such as *MBTD1::EZHIP* [62,64,66]. Of note, *MEAF6::PHF1* has also been identified in ESNs, again highlighting the biological continuum between these lesions [62,67].

Recent RNA sequencing studies have expanded the molecular spectrum of LG-ESS, revealing novel gene fusions such as *RNF111::ARID2*, *ESR1::NCOA3*, *PTCH1::GLI1*, *PHF21A::NFIA*, *PHF21A::CETP*, *ACTB::GLI1*, and *GREB1::NCOA2*. While the biological roles of these rare rearrangements remain to be clarified, they likely affect chromatin remodeling, transcriptional regulation, and hormone receptor signaling pathways [62,64,66].

Clinically, LG-ESS behaves as an indolent but recurrent neoplasm with characteristic fusion-driven biology that separates it from ESN on one end of the spectrum and high-grade sarcomas on the other [57,58,60,64,66].

### 3.3. High-Grade Endometrial Stromal Sarcoma (HG-ESS)

HG-ESS is a rare, aggressive uterine stromal tumor occupying an intermediate position between LG-ESS and UUS. Histologically, HG-ESS demonstrates greater nuclear atypia, increased mitotic activity, and variable architectural patterns, ranging from diffuse sheets of tumor cells to fascicular or nested arrangements. Some tumors exhibit biphasic morphology, containing both low- and high-grade components [58,59,60,61,62,63,68].

HG-ESS is defined by distinct, recurrent gene fusions, each associated with characteristic morphologic and immunophenotypic profiles [66,68].

The most well-characterized subtype harbors the *YWHAE::NUTM2A/B* [t(10;17)(q22;p13)] fusion, typically associated with round to epithelioid tumor cells, brisk mitotic activity, and strong Cyclin D1 expression and often diffuse BCOR expression despite lack of *BCOR* gene alteration. These tumors are usually negative for estrogen and progesterone receptors, in contrast to LG-ESS. Morphologically, they often contain sheets of intermediate-sized round to ovoid cells with open chromatin and scant to moderate eosinophilic cytoplasm, frequently juxtaposed to spindle cell areas resembling fibroblastic LG-ESS [66,68,69,70].

A second major molecular subtype is defined by the *ZC3H7B::BCOR* fusion, identified through NGS. Retrospective analyses have shown *ZC3H7B* to be the most frequent *BCOR* fusion partner, though multiple others have been described, including genes involving transcriptional regulation (*EP300* and *CREBBP*) and chromatin modification (*NUTM2G*, *L3MBTL2*, and *KMT2D*) [71]. Tumors with *BCOR* rearrangements often show fascicular pattern or relatively uniform spindle cells, variable atypia, and focal myxoid stroma. Frequent Cyclin D1 overexpression is present due to activation of the cyclin D1-CDK4 pathway, often accompanied by *MDM2* amplification or *CDKN2A* deletion. BCOR expression present in approximately 50% cases [66,68,69,70,71].

A subset of HG-ESS lacks gene fusion but harbors *BCOR* internal tandem duplications (ITDs) involving exon 15, resulting in duplication of the C-terminal region of the BCOR protein. These tumors share morphologic and immunophenotypic similarities with *BCOR* rearrangement cases and may harbor *MDM2* amplifications [66,68,69,70,71,72].

As a core component of a variant PRC1, BCOR modifies to silence or suppress the activity of target genes, including *HOX* genes, which are critical for embryonic development and cell differentiation. In normal conditions, BCOR acts as a tumor suppressor. Its altered function contributes to tumor progression, impacting cell proliferation and epigenetic regulation, and is associated with a poor prognosis [73]. BCOR expression is a key diagnostic biomarker in HG-ESS (Table 1).

Collectively, broad genetic landscape of HG-ESS indicate that these tumors are genetically heterogeneous, and the involved genes often converge on a common oncogenic mechanism, primarily the dysregulation of gene transcription through effects on chromatin remodeling complexes (e.g., *BCOR*, *KDM2B*, *EPC1*, *BRD8*), signaling and proliferation pathways (e.g., *YWHAE*, *NUTM2*, *EML4*, *NTRK1*, *NTRK3* and *STRN*), structural and transport pathways (e.g., *TPR*, *LMNA*, *TPM3*) and gene regulation pathways (e.g., *ZC3H7B*, *RBPMS*, and *COL1A*). The increasing use of NGS has greatly enhanced recognition of this heterogeneity and clarified overlaps with other uterine sarcoma subtypes [66,68,69,74].

Although the precise mechanisms by which these genetic events drive oncogenesis remain under investigation, accumulating evidence implicates dysregulation of chromatin remodeling and transcriptional control as central pathogenic mechanisms. As sequencing technologies continue to evolve, additional driver alterations are likely to be identified, further refining diagnostic criteria and potentially informing targeted therapeutic approaches in HG-ESS.

### 3.4. Undifferentiated Uterine Sarcoma (UUS)

UUS is a highly aggressive uterine mesenchymal neoplasm that lacks morphologic and immunophenotypic evidence of specific cell differentiation. Formerly termed undifferentiated endometrial sarcoma, UUS may arise from either the endometrium or myometrium [58,60,74]. Histologically, these tumors are composed of markedly atypical cells arranged in sheets, storiform, or herringbone patterns. Areas of rhabdoid morphology or myxoid stroma may also be seen. Hallmark features include destructive myometrial invasion, brisk mitotic activity, tumor necrosis, and frequent vascular invasion. Although clinical data are limited, the prognosis is generally poor, reflecting the tumor’s high-grade biological behavior [75].

The diagnosis of UUS is essentially one of exclusion, made only after more common uterine sarcomas, particularly leiomyosarcoma and ESS, have been ruled out [75,76]. Notably, recent molecular studies suggest that many tumors historically diagnosed as UUS are, in fact, HG-ESS that were not recognized as such prior to the identification of defining gene fusions. In this context, BCOR immunoexpression in ≥50% of tumor cells can serve as a useful screening tool for selecting cases for molecular testing to confirm HG-ESS [71,75].

True UUS cases, those without evidence of ESS-related fusions, appear to constitute a molecularly heterogeneous group. Some exhibit *SMARCA4* deficiency or *TP53* mutations, as reported in a 2020 NIH study [77,78], while others lack these alterations but do not demonstrate known ESS-related fusions such as *JAZF1* or *NTRK*, which are used diagnostically to exclude other specific sarcoma types [68,74]. Emerging evidence suggests that a subset of UUS harbors distinct gene expression signatures, with one subgroup showing enrichment of genes involved in muscle cell development pathways, hinting at divergent differentiation potential.

In a recent study by Dundr et al., which analyzed 74 UUS cases, partial overlap in the mutation profile was observed between UUS and monomorphic HG-ESS lacking recurrent fusions (nf_HG-ESS) [79]. These findings further support the notion that UUS is not a single entity but rather a heterogeneous collection of tumors that may include unrecognized molecular subsets. However, due to the rarity of these neoplasms, data correlating molecular subtypes with clinical outcomes remain limited. Another recent unsupervised molecular clustering studies of UUS have identified several distinct subgroups. One cluster displayed a molecular profile closely resembling fusion-negative HG-ESS, while another showed strong similarity to muscle cell-differentiated tumors [80]. These findings suggest that UUS likely represents a spectrum of biologically distinct, undifferentiated uterine sarcomas, unified more by the absence of defining differentiation than by a single shared molecular pathogenesis.

Morphologic and immunohistochemical distinctions between HG-ESS and UUS are often subtle and somewhat arbitrary. Molecular testing has therefore become an invaluable adjunct for accurate classification. Nonetheless, in cases with equivocal morphologic features and absence of characteristic genetic aberrations, the differentiation between HG-ESS and UUS remains challenging and occasionally subjective based on current diagnostic criteria [63,66,69,75].

## 4. Uterine Perivascular Epithelioid Cell Neoplasms (PEComas)

PEComas are rare mesenchymal neoplasms characterized by a distinctive cell type showing dual myogenic and melanocytic differentiation [81,82,83]. Initially described in the early 1990s and formally recognized by the World Health Organization in 2002, PEComas encompass a family of tumors that includes angiomyolipoma, lymphangioleiomyomatosis, and related entities [81]. Within the gynecologic tract, the uterus is the most frequently affected site, although these tumors remain uncommon and pose diagnostic and management challenges due to their heterogeneous morphology and variable clinical behavior [82,83].

Histologically, uterine PEComas are composed of epithelioid to spindle cells arranged around blood vessels in nests, sheets, or trabeculae. Variable mitotic activity, necrosis, and nuclear atypia may be present in more aggressive examples. Immunohistochemically, PEComas coexpress smooth muscle markers (SMA, desmin, and caldesmon) and melanocytic markers (HMB45 and melan-A), which form the basis for diagnosis [82,83].

Molecular alterations in uterine PEComas frequently involve dysregulation of the mTOR signaling pathway, most often due to inactivating mutations in the *TSC1* or *TSC2* genes. These mutations result in constitutive mTOR pathway activation, promoting tumor cell growth and proliferation, and providing a rational target for mTOR inhibitor therapy [83,84]. Loss of ATRX expression, associated with *ATRX* gene mutations, is observed in a subset of cases and may correlate with more aggressive behavior [84,85]. Mutations in *BRCA2* have also been reported in a minority of PEComas [84,86].

A distinct molecular subset of uterine PEComas harbors *TFE3* gene rearrangements, leading to overexpression of the TFE3 transcription factor. These *TFE3*-rearranged PEComas tend to occur in younger patients and often exhibit epithelioid morphology, high nuclear grade, and a more aggressive clinical course [84,86,87]. Detection of TFE3 overexpression by immunohistochemistry should prompt confirmatory molecular testing, such as FISH or RNA sequencing, to identify *TFE3* fusions (Table 1) [84,86,87].

**Figure 3:**
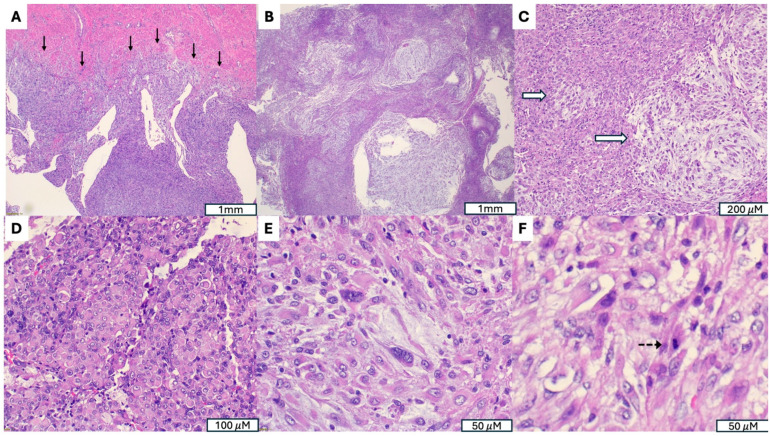
Malignant Perivascular Epithelioid Cell Tumor (PEComa) with *TFE3* Rearrangement. (**A**) Infiltrative growth into the myometrium (indicated by →). (**B**) Tumor exhibits a multinodular architecture composed of poorly circumscribed nodules with irregular infiltrative borders, separated by variably myxoid stroma. (**C**) Myxoid stroma (indicated by ⇒). (**D**) Areas with dense eosinophilic, rhabdoid appearing cytoplasm. (**E**) Marked cytological atypia, with severe nuclear pleomorphism, irregular hyperchromatic nuclei and prominent nucleoli. (**F**) Increased mitotic activity (indicated by -->).

Clinically, a subset of uterine PEComa demonstrates malignant potential, with local recurrence or distant metastasis. Morphologic and molecular parameters associated with poor outcome include large tumor size, high mitotic index, necrosis, nuclear atypia, infiltrative growth, and the presence of *TFE3* rearrangements (Figure 3) [87,88]. Surgical excision remains the primary treatment modality, while patients with advanced or recurrent disease may benefit from targeted therapy with mTOR inhibitors such as sirolimus or everolimus, particularly in tumors with *TSC1* or *TSC2* alterations (Table 2) [82,89].

## 5. Uterine Inflammatory Myofibroblastic Tumors (IMTs)

Uterine inflammatory myofibroblastic tumors (IMTs) are rare mesenchymal neoplasms characterized by a proliferation of spindle-shaped myofibroblastic cells admixed with variable lymphoplasmacytic inflammation. Initially described in the lungs, IMTs have been identified in multiple anatomic sites, including the female genital tract [90,91]. Within the uterus, IMTs are uncommon and often clinically or morphologically mistaken for leiomyomas due to overlapping features [92]. IMTs of the uterus are often associated with pregnancy and are delivered with the placenta [93]. Grossly, they tend to have a softer, gelatinous texture and may show ill-defined or irregular borders [90,91].

**Figure 4:**
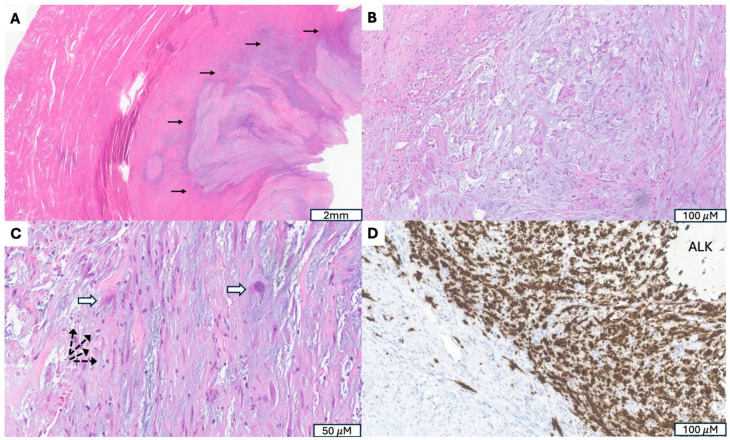
Inflammatory Myofibroblastic Tumor with *ALK* Gene Fusion. (**A**) Irregular, infiltrative tumor border (indicated by →). (**B**) Fasciitis-like proliferation of spindle cells in a myxoid, hypocellular background. (**C**) Presence of ganglion-like tumor cells (indicated by ⇒) accompanied by scattered lymphoplasmacytic inflammation (indicated by -->). (**D**) Granular cytoplasmic ALK immunostaining highlighting tumor cells.

Microscopically, uterine IMTs exhibit diverse growth patterns, ranging from myxoid and vascular to compact spindle cell or hypocellular fibrous areas, often accompanied by a chronic inflammatory infiltrate. Mitotic activity is usually low, although occasional atypical mitoses and necrosis can be seen in more aggressive examples (Figure 4) [90,91]. Immunohistochemically, IMTs express smooth muscle markers such as SMA, desmin, and caldesmon, as well as CD10. ALK expression, resulting from *ALK* gene rearrangement, is observed in majority uterine IMTs and is considered a key diagnostic feature (Figure 4, Table 1) [90,91,92,94].

Molecularly, up to 90% uterine IMTs harbor *ALK* rearrangements, in which the 3′ portion of *ALK* containing the tyrosine kinase domain fuses with the 5′ end of a highly expressed partner gene, leading to constitutive kinase activation. Reported fusion partners include *THBS1*, *TIMP3*, *IGFBP5*, *FN1*, *NRP2*, *DES*, *SEC31*, *TNS1*, *CASC15*, and *TPM3*. A rare, more aggressive variant, epithelioid inflammatory myofibroblastic sarcoma (EIMS), is associated with *RANBP2::ALK* or *RRBP1::ALK* fusions and is characterized by epithelioid morphology and poor clinical outcomes. Pregnancy-associated uterine IMTs frequently harbor *TIMP3::ALK* or *THBS1::ALK* fusions, suggesting a potential hormonal or gestational influence in their pathogenesis [91,95,96].

Approximately 10% of uterine IMTs lack *ALK* rearrangements and instead harbor alternative kinase fusions involving *ROS1*, *RET*, *PDGFRB*, or *NTRK3*. Reported examples include *TIMP3::RET*, *SORBS1::RET*, *TIMP3::ROS1*, *FN1::ROS1*, *NUDCD3::ROS1*, *TFG::ROS1*, *IGFBP5::PDGFRB*, *THBS1::INSR*, and *ETV6::NTRK3*. In ALK-negative cases, RNA-based NGS can aid in identifying these rearrangements [91,95,96,97,98].

Most uterine IMTs behave in a benign or locally recurrent manner, but tumors with cytologic atypia, necrosis, LVI, or increased mitotic activity may show more aggressive clinical behavior [95,96]. Complete surgical excision with negative margins is the mainstay of management. For recurrent, unresectable, or metastatic disease, targeted therapy with ALK inhibitors such as crizotinib, ceritinib, or alectinib has demonstrated clinical efficacy (Table 2). In ALK-negative tumors with alternative kinase fusions, targeted agents directed against ROS1, RET, or NTRK may be beneficial [96,97,98,99].

## 6. Uterine Tumors Resembling Ovarian Sex Cord Tumor (UTROSCT)

**Figure 5:**
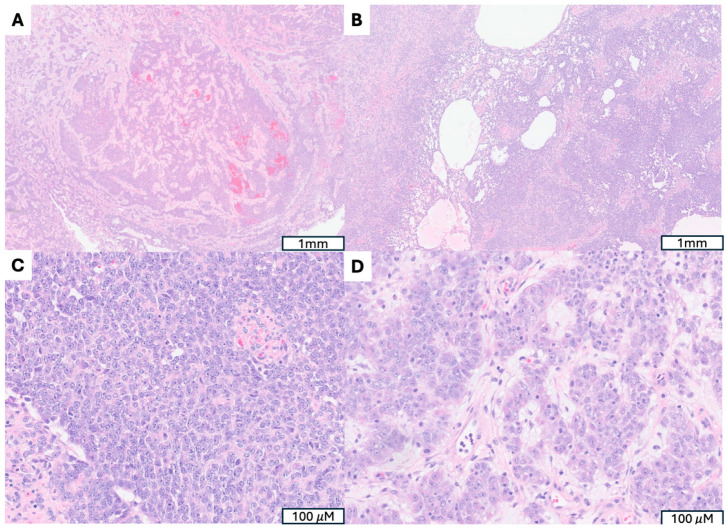
A Uterine Tumors Resembling Ovarian Sex Cord Tumor with Confirmed *GREB1::NCOA2* Fusion. (**A**,**B**) Marked architectural diversity, including trabecular and nested arrangement (**A**) as well as cystic, glandular and solid patterns (**B**). (**C**) Epithelioid tumor cells mimic granulosa cell tumor. (**D**) Polygonal cells with abundant eosinophilic granular cytoplasm and prominent nucleoli, resembling Leydig cell tumor.

Uterine tumor resembling ovarian sex cord tumor (UTROSCT) is a rare uterine mesenchymal neoplasm, with approximately 500 cases reported to date [100,101]. The number of reported cases has been increasing in recent years, largely due to advances in immunohistochemical and molecular characterization that have improved diagnostic accuracy. In the 2020 World Health Organization (WHO) classification of female genital tumors, UTROSCT is categorized under miscellaneous mesenchymal tumors, reflecting its uncertain histogenesis [1,100,101,102]. Morphologically, UTROSCTs exhibit remarkable architectural diversity, displaying trabecular, cord-like, glandular, tubular, or nested patterns that closely mimic ovarian sex cord tumors. The tumor cells are typically epithelioid to spindled, with moderate cytoplasm and mild to moderate nuclear atypia. The stroma may be fibrous or hyalinized, and mitotic activity is generally low (Figure 5). Certain features, such as infiltrative margins, cytologic atypia, necrosis, or LVI, may correlate with more aggressive clinical behavior [103,104].

UTROSCTs frequently pose diagnostic challenges due to their morphologic overlap with a wide range of other uterine neoplasms, including LG-ESS, cellular or epithelioid leiomyoma, PEComa, and endometrial carcinoma. Immunohistochemistry plays a central role in diagnosis, with UTROSCTs showing a polyphenotypic expression profile that reflects their mixed differentiation. These tumors variably express sex cord–stromal markers such as calretinin, inhibin, SF1, FOXL2, CD99, and CD56, together with epithelial markers (AE1/3, EMA), smooth muscle markers (desmin, SMA), and hormone receptors (ER, PR). Because no single marker is specific, an immunohistochemical panel incorporating at least two markers from each lineage improves diagnostic reliability [100,102,104].

Molecular studies have clarified that recurrent gene fusions involving members of the nuclear receptor coactivator (*NCOA*) family, specifically *NCOA1*, *NCOA2*, and *NCOA3*, represent the defining molecular feature of UTROSCT. The most frequent fusion event is *ESR1::NCOA3*, followed by *GREB1::NCOA2*, whereas other fusions such as *GREB1::CTNNB1*, *GTF2A1::NCOA2*, and *ESR1::CITED2* have also been described [101,102]. These genetic findings distinguish UTROSCT from LG-ESS and other uterine mesenchymal neoplasms (Table 1). Recent large-scale genomic profiling has demonstrated that tumors harboring *GREB1* or *NCOA2-related* fusions cluster separately from those with *ESR1::NCOA3* fusions or without detectable fusions, suggesting that molecular subgroups may exist with distinct biological and potentially prognostic implications [74,103,104,105].

Clinically, most UTROSCTs behave in an indolent manner, with approximate 10–20% subset exhibiting recurrence or metastasis [100,101]. Adverse prognostic factors include large tumor size, high mitotic activity, cytologic atypia, necrosis, LVI, and infiltrative growth. Although prognostic correlations with specific fusion types remain under investigation, early evidence suggests that certain genetic subtypes such as *GREB1::NCOA2* fusion may confer greater risk of aggressive clinical behavior [103,104,105]. Integrating morphologic, immunophenotypic, and molecular findings is therefore critical for accurate classification and prognostication. While there are currently no targeted therapies specific to UTROSCT, the expanding understanding of its molecular landscape is likely to inform future diagnostic criteria and therapeutic strategies.

## 7. Emerging Molecularly Defined Uterine Mesenchymal Tumors

### 7.1. NTRK-Rearranged Uterine Sarcomas

*NTRK*-rearranged uterine sarcomas are rare spindle cell neoplasms that predominantly arise in the uterine cervix of young or premenopausal women, though cases in children and older adults have been reported [106]. These tumors are driven by fusions involving *NTRK1*, *NTRK2*, or *NTRK3* with various partner genes, producing constitutively active TRK fusion proteins that promote tumor growth [106,107,108,109,110].

Originally described by Chiang et al. in 2018, *NTRK* rearranged uterine sarcomas typically display fibrosarcoma-like spindle cell morphology, often arranged in fascicles with minimal pleomorphism, although occasional symplastic or atypical foci may be present [111,112]. Immunohistochemically, all reported tumors are positive for pan-TRK, with variable expression of S100 and CD34 [106,108,111]. Molecular studies have identified *NTRK1* fusions in the majority of cases and *NTRK3* fusions in a subset, with partner genes including *TPR*, *TPM3*, *EML4*, *TFG*, *SPECC1L*, *C16orf72*, and *IRF2BP2*. Rare cases with unusual morphology, sometimes initially classified as unclassifiable uterine sarcomas, have also harbored *TP53* mutations [106,108,111,112].

Clinically, *NTRK*-rearranged uterine sarcomas demonstrate variable behavior. While some tumors recur or metastasize, consistent morphologic or molecular predictors of prognosis have not yet been established. Proposed adverse features include a mitotic index of ≥8 per 10 high-power fields, LVI, necrosis, and the presence of an *NTRK3* fusion [106,110]. Tumors lacking these characteristics generally have an excellent prognosis [106,110]. Accurate diagnosis is critical, Recognition of this entity is clinically important, as the presence of an actionable *NTRK* fusion allows treatment with targeted TRK inhibitors such as larotrectinib or entrectinib, for patients with advanced or metastatic disease (Table 2) [109,110,112].

### 7.2. COL1A1::PDGF Fusion Uterine Sarcoma

Uterine sarcomas are rare neoplasms, some of which are defined by characteristic gene fusions. Among them, *COL1A1::PDGFB* fusion uterine sarcoma is a recently described entity that shares the same genetic alteration as dermatofibrosarcoma protuberans (DFSP) [113,114,115]. Its high-grade variant remains poorly characterized. While DFSP typically arises in the skin and superficial soft tissues, its occurrence in visceral organs, particularly the uterus, is exceedingly rare and may pose diagnostic challenges due to morphologic overlap with other uterine spindle cell neoplasms. To date, less than 20 cases of uterine sarcoma with *COL1A1::PDGFB* fusion have been reported [113,114,115], including one high-grade case harboring both the *COL1A1::PDGFB* fusion and a pathogenic *TP53* (p.P278A) mutation [114].

### 7.3. MEIS1::NCOA2/1 Fusion Sarcoma

*MEIS1::NCOA2/1* fusion sarcoma is a rare, recently recognized spindle cell neoplasm defined by a characteristic gene fusion involving *MEIS1* and *NCOA2* or, less commonly, *NCOA1*. These tumors most frequently arise in the genitourinary tract, including the kidneys, and in gynecologic organs such as the uterus and vagina, though rare cases have been reported at other sites [116,117,118,119].

Histologically, tumors are composed of plump spindle cells arranged in short fascicles, often demonstrating an infiltrative growth pattern. They exhibit nonspecific morphologic features and a variable immunoprofile, with inconsistent expression of markers such as ER, PR, CD10, cyclin D1, TLE1, and WT1. This nonspecific appearance can closely mimic other spindle cell neoplasms, particularly ESS, making molecular confirmation via NGS essential for accurate diagnosis [116,118,119,120].

Clinically, most *MEIS1::NCOA2*/*1* fusion sarcomas behave as low-grade malignant tumors, showing a tendency for local recurrence but a low risk of distant metastasis or disease-related death. However, a subset displays aggressive behavior, with high-grade morphology and potential for metastases, including to the lung. Aggressive cases may harbor additional genetic alterations beyond the defining fusion, such as amplification of genes on chromosome 12q13–15 (*MDM2*, *CDK4*, *MDM4*) or mutations in *CTNNB1*, which may contribute to tumor progression and high-grade transformation [119].

Because of their heterogeneous morphology, nonspecific immunophenotype, and varied anatomic distribution, *MEIS1::NCOA2/1* fusion sarcomas can be misdiagnosed as other spindle cell neoplasms. Molecular testing is therefore critical for correct classification, prognostication, and guiding clinical management.

### 7.4. SMARCA4-Deficient Uterine Sarcoma (SDUS)

SMARCA4-deficient uterine sarcoma (SDUS) is a rare and highly aggressive uterine malignancy defined by the inactivation or loss of the SMARCA4 gene, a key component of the SWI/SNF chromatin remodeling complex [3,121,122]. It typically affects young women and carries a poor prognosis, with a reported median survival of approximately seven months [77,121].

**Figure 6:**
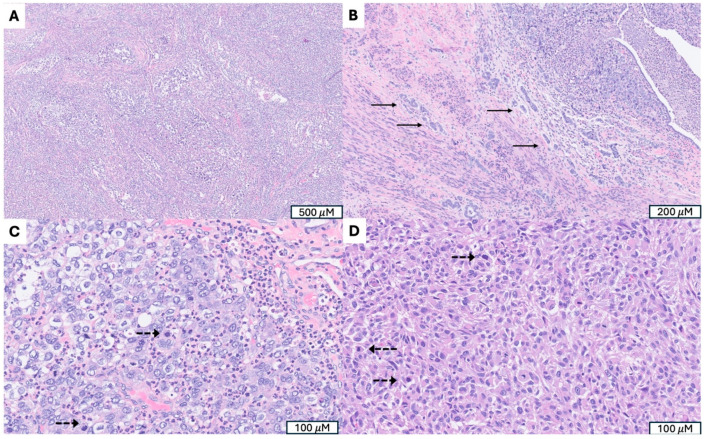
SMARCA4-deficient uterine sarcoma (SDUS). (**A**) Sheets of poorly differentiated tumor cells. (**B**) Extensive lymphovascular invasion (indicated by →). (**C**) Marked cytological atypia with prominent nucleoli and frequent mitosis (mitotic figures indicated by -->). (**D**) Rhabdoid tumor cells with abundant eosinophilic cytoplasm and eccentric nuclei, a hallmark morphologic feature of SDUS.

Histologically, SDUS is characterized by sheets of poorly differentiated rhabdoid cells with abundant eosinophilic cytoplasm, eccentric nuclei, and prominent nucleoli. The tumors frequently exhibit extensive LVI and a high propensity for extrauterine spread at presentation (Figure 6). Immunohistochemically, there is loss of SMARCA4 (BRG1) protein expression, confirming the underlying genetic alteration, while other markers are usually nonspecific [77,78,122,123].

Molecularly, SDUS is driven by *SMARCA4* inactivation and tends to lack other recurrent genetic alterations. It shares morphological, molecular, and clinical features with other SMARCA4-deficient neoplasms, such as SMARCA4-deficient thoracic sarcoma and small cell carcinoma of the ovary, hypercalcemic type (SCCOHT), suggesting a spectrum of related tumors linked to SWI/SNF complex dysfunction [77,78,121,122,123].

Clinically, SDUS is an aggressive neoplasm with rapid progression and poor outcome despite multimodal therapy. Research into potential targeted treatments is ongoing. Emerging evidence suggests that SDUS may show sensitivity to immunotherapy, and preclinical studies have indicated potential efficacy of EZH2 and CDK4/6 inhibitors (Table 2) [3,121,124]. Some cases have been associated with germline SMARCA4 mutations, raising concern for rhabdoid tumor predisposition syndrome (RTPS) and highlighting the importance of genetic counseling for affected patients and their families [125,126].

### 7.5. RAD51B Fusion Uterine Sarcoma

*RAD51B* fusion uterine sarcoma is not a morphological diagnosis, but rather a mixture of recently recognized subset of uterine sarcomas characterized by gene fusions involving *RAD51B* and various partner genes, most commonly *HMGA2* or *NUDT3* [127,128]. These fusions have been identified across a spectrum of uterine mesenchymal neoplasms, including PEComas, leiomyosarcomas, and UUS, suggesting that *RAD51B* rearrangement may represent a shared molecular event contributing to tumorigenesis [80,127,128,129].

These tumors arise predominantly in the uterus and display considerable morphologic heterogeneity, often exhibiting overlapping histologic features with other uterine spindle cell neoplasms [128]. Clinically, *RAD51B* fusion uterine sarcomas tend to behave aggressively and are associated with an unfavorable prognosis [127,128,129]. With the increasing use of next-generation sequencing, recognition of *RAD51B*-rearranged uterine sarcomas has grown, and they are now regarded as a distinct molecular subgroup of uterine sarcomas with diverse morphologic manifestations but a typically malignant clinical course [128].

### 7.6. KATB/A::KANSL1 Fusion Sarcoma

*KAT6B/A::KANSL1* fusion sarcoma is a recently recognized and distinct subtype of uterine sarcoma defined by a characteristic gene fusion involving *KAT6B* or, less commonly, *KAT6A* and *KANSL1* [79,130,131,132]. This recurrent molecular alteration leads to the formation of an abnormal fusion protein that drives tumorigenesis. Although these tumors may display deceptively bland or low-grade histologic features, they often behave in an aggressive manner [130,131,132,133,134].

Histologically, *KAT6B/A::KANSL1* fusion sarcomas exhibit overlapping morphologic features with both endometrial stromal and smooth muscle tumors, sometimes with sex cord-like pattern, which can make diagnosis challenging. The neoplasm may appear relatively low-grade under the microscope, yet many cases demonstrate aggressive clinical behavior with potential for recurrence and metastasis [130,131].

This entity has been established as a distinct clinicopathologic category, separate from LG-ESS and leiomyoma, with which it was previously misclassified [132,133,134]. Given its hybrid morphology and nonspecific immunophenotype, definitive diagnosis requires molecular confirmation through NGS or FISH to detect the *KAT6B/A::KANSL1* gene fusion. More comprehensive work-up, including CNV, methylation and mutational analysis, can yield additional prognostic information when a *KAT6B/A::KANSL1* is identified in a uterine mesenchymal neoplasm [131].

### 7.7. ERBB2/3 Mutated S100/SOX10-Positive Uterine Sarcoma

S100/SOX10-positive uterine sarcoma with *ERBB2*/*ERBB3* mutation is a newly recognized and highly aggressive subtype of uterine sarcoma characterized by aberrant HER2 pathway activation and neural crest-like immunophenotype [135,136,137]. These tumors are defined by activating mutations or amplifications involving *ERBB2* or *ERBB3*, leading to overexpression of the HER2 protein and downstream oncogenic signaling.

Histologically, they are composed of high-grade spindle and/or round cells with variable pleomorphism, prominent nucleoli, and brisk mitotic activity. Immunohistochemically, the tumor cells show diffuse positivity for S100 and SOX10, markers typically associated with melanocytic or peripheral nerve sheath differentiation, while lacking expression of conventional smooth muscle or endometrial stromal markers [135,136,137].

Additional molecular alterations, including *ATRX* and *CDKN2A* mutations, have been reported in a subset of cases [135,136,137]. Clinically, these tumors exhibit an aggressive course with frequent local recurrence and distant metastasis [135,137]. Recognition of this entity is important for accurate classification and therapeutic stratification, as HER2-targeted therapies may offer potential benefit in tumors harboring *ERBB2* amplification or activating mutations (Table 2).

## 8. Timing of Sampling and Stage-Dependent Detection of Molecular Alterations

The timing of tissue sampling plays an important role in the molecular characterization of uterine mesenchymal tumors, although available data remain limited and vary by tumor subtype. In general, fusion-driven neoplasms, including *JAZF1*-rearranged LG-ESS, *YWHAE-* or *BCOR*-altered HG-ESS, *ALK*- or *NTRK*-rearranged IMTs, and *TFE3*-rearranged PEComas, harbor early, lineage-defining genomic events that are consistently detectable across disease stages. These alterations appear to represent primary oncogenic drivers and are not typically stage-dependent. Consequently, molecular testing performed on either initial biopsy or resection specimens is usually sufficient for accurate classification [138].

By contrast, accumulating evidence suggests that genetic complexity increases with tumor progression in certain high-grade sarcomas, particularly uterine leiomyosarcoma. Studies have shown that while early lesions may demonstrate a limited set of canonical alterations (e.g., *TP53*, *RB1*, *ATRX*), advanced-stage or recurrent tumors exhibit greater genomic instability, higher mutational burden, and additional copy-number alterations, which may influence both prognosis and therapeutic responsiveness [138].

Overall, evidence shows that lineage-defining driver fusions remain stable across disease stages, while secondary mutations linked to genomic instability and treatment resistance emerge more often in advanced tumors. Thus, molecular testing at initial diagnosis is typically sufficient for accurate classification, with repeat testing at recurrence reserved for identifying newly actionable alterations.

## 9. Integrating Molecular Insights into Future Diagnostic and Therapeutic Strategies

Recent advances in molecular diagnostics have reshaped the understanding of uterine mesenchymal tumors by revealing biologically distinct subgroups defined by specific oncogenic drivers. Rather than simply confirming histologic impressions, molecular profiling has demonstrated that tumors with similar morphology may arise from divergent molecular lineages and exhibit markedly different clinical behaviors. For example, SDUS is now recognized as a highly aggressive, standalone entity that cannot be reliably distinguished from undifferentiated sarcoma on morphologic grounds [77,80]. Likewise, *YWHAE*-rearranged high-grade ESS represents a molecularly distinct category separate from LG-ESS despite some histologic overlap [63,66]; and *NTRK*-rearranged uterine spindle cell sarcomas form a fusion-defined group with a characteristic immunophenotype and a clear therapeutic target not apparent on routine histology [108,113].

Accordingly, incorporating molecular testing into diagnostic workflows extends far beyond confirmatory utility: it enables identification of clinically actionable genomic alterations, refines prognostic stratification, and guides targeted therapeutic selection. These benefits are reflected in the expanding catalog of actionable pathways in uterine sarcomas (Table 1). Future multi-omics efforts will be critical to clarifying tumor ontogeny, defining molecular transitions underlying progression, and uncovering additional therapeutic vulnerabilities that can serve as the basis for rational, biology-driven management.

The molecular diversity of uterine mesenchymal tumors translates into multiple targetable pathways (Table 2). For example, in uterine leiomyosarcoma, alterations affecting HRD may confer sensitivity to PARP inhibition [46], while MMR-deficient tumors may respond to immune checkpoint blockade [45,46]. Low-grade ESS characterized by *JAZF1::SUZ12* and related fusions may be susceptible to agents targeting chromatin remodeling or Wnt signaling, complementing established hormonal therapies [65]. Rare entities also exhibit targetable vulnerabilities. PEComas with *TSC1/2* mutations or *TFE3* rearrangements respond to mTORC1 inhibition [83,87,89], while IMTs harboring *ALK*, *RET*, *PDGFRB*, or *NTRK* rearrangements demonstrate marked responses to ALK or TRK inhibitors [90,106,112]. SDUS may be susceptible to EZH2 or CDK4/6 inhibition [78,122], and early evidence suggests that ERBB2/3-mutated S100/SOX10-positive sarcomas may benefit from HER2-targeted therapy [136]. Collectively, these findings underscore the rapidly expanding role of precision oncology in the management of uterine mesenchymal tumors.

Molecular profiling has redefined uterine mesenchymal tumors by revealing distinct, driver-defined subgroups that enhance diagnostic accuracy and increasingly guide targeted therapy. Moving forward, integrating early, and when clinically indicated, repeating molecular assessment will be essential to capture both stable lineage-defining events and newly emergent actionable alterations. Yet important gaps remain: many rare tumors lack robust genomic characterization, mechanisms of progression and therapeutic resistance are poorly understood, and functional preclinical models remain limited. Addressing these challenges will require next-generation approaches, including spatial transcriptomics to resolve intratumoral heterogeneity and tumor-microenvironment interactions, as well as evolutionary genomic mapping to trace tumor development back to putative stem or progenitor cells and delineate clonal trajectories that give rise to aggressive subpopulations. These efforts will be critical for refining molecular taxonomy and enabling truly adaptive, biology-driven management of this heterogeneous family of neoplasms.

## Figures and Tables

**Table 1:** Uterine Mesenchymal Tumors Molecular Landscape and Potential Diagnostic and Prognostic Utility.

Category	Entity	Variants	Molecular Pathology	Diagnostic Utility	Prognostic Utility
Smooth muscle tumors	Uterine leiomyoma		*MED12* mutation, *HMGA2* and *HMGA1* fusion, *COL4A5* and *COL4A6* deletion FH mutation	N/A	*FH-deficient* leiomyoma prompt genetic consulting for HLRCC syndrome
	Intravenous leiomyomatosis		*HMGA2* gene fusion	N/A	N/A
	Uterine leiomyosarcoma	Spindle (conventional)	*TP53*, *RB1*, *ATRX*, *PTEN*, *CDKN2A*, *MDM2* mutations	A molecular-based IHC algorithm for diagnosis is proposed	NA
		Epithelioid	*PGR* gene fusions	N/A	Aggressive subset
		Myxoid	*PLAG1* gene fusion	Maybe helpful	N/A
Endometrial stromal tumors	Endometrial stromal nodule		some harbor fusions found in LG-ESS, such as *JAZF1-SUZ12*	N/A	N/A
	LG ESS		*JAZF1::SUZ12*, *JAZF1::PHF1*, *PHF1* gene fusions	Maybe helpful	N/A
	HG ESS	*YWHAE::NUTM2A/B*	*YWHAE::NUTM2A/B*	Helpful in diagnosis especially in HG-ESS with LG features	N/A
		*BCOR* gene fusion	*ZC3H7B::BCOR*, *BCOR* with other partners		N/A
		*BCOR* ITD			N/A
	Undifferentiated uterine sarcoma	a heterogeneous collection includes unrecognized molecular subsets			N/A
Miscellaneous	Uterine tumor resembling ovarian sex cord tumors (UTROSCT)		*ESR1::NCOA3*, *GREB1::NCOA2*, other *NCOA* family gene fusion		*GERB1::NCOA2* may confer aggressive behavior
	Perivascular epithelioid cell tumor (PEComa)	*TSC1* or *TSC2* gene mutation			N/A
		*TFE3* gene rearrangement		Helpful	More aggressive
	Inflammatory myofibroblastic tumor (IMT)		*ALK* rearrangement most common; *RPS1*, *RET1*, *PDGFRB* or *NTRK3* rearrangement	ALK IHC	N/A
Emerging molecularly defined uterine mesenchymal tumors	*NTRK*-rearranged uterine sarcomas		Molecularly defined entity		Bland morphology, variable behavior
	*COL1A1::PDGF* fusion uterine sarcoma				Most low-grade
	*MEIS1::NCOA2/1* fusion sarcoma				Behave as low-grade sarcoma
	SMARCA4-deficient uterine sarcoma (SDUS)				Worse outcome
	*RAD51B* fusion uterine sarcoma				Aggressive
	KATB/A::KANSL1 fusion sarcoma				Poor prognosis
	ERBB2/3 mutated S100/SOX10-positive uterine sarcoma				N/A

Abbreviates: N/A: Not applicable

**Table 2:** Targetable Molecular Pathways and Emerging Therapeutic Approaches.

Entity	Potential Targetable Molecular Alteration	Affected Pathways	Targeted Therapy
Uterine leiomyosarcoma	*BRCA2*, *RAD51B*, *PALB2*	DNA repair-HRD	PARP inhibitor
	MMR deficient	DNA repair-MMR	PD-L1
	*PTEN*	PI3K/mTOR/AKT	CDK4/6 inhibitor mTOR inhibitor
	*PGR* fusion	Estrogen-driven signaling	Hormone therapy
Low-grade endometrial stromal sarcoma	*JAZF1::SUZ12*, and other fusion	Chromatin remodeling/Wnt pathway	Wnt pathway inhibitor
High-grade endometrial stromal sarcoma	*YWHAE::NUTM2A/B*	RAF/mitogen-activated protein kinase and Hippo pathways, with downstream cyclin D1	CDK4/6 inhibitor Mitogen kinase inhibitor
	*BCOR*-rearranged or *ITD*	cyclin D-CDK4/6-Rb pathway MDM2 amplification	CDK4/6 inhibitor MDM2 inhibitors
	*NTRK1/NTRK3* rearrangements	TRK pathway activation	TRK inhibitor
Perivascular epithelioid cell tumor (PEComa)	*TSC1/2* mutation *TFE3* rearrangement	mTOR pathway	mTORC1 inhibitor
Inflammatory myofibroblastic tumor (IMT)	*ALK*, *RPS1*, *RET1*, *PDGFRB* or *NTRK* rearrangement	ALK and other tyrosine kinase pathways	ALK inhibitor
NTRK-rearranged uterine sarcomas	*NTRK*-rearrangement	TRK pathway activation	TRK inhibitor
SMARCA4-deficient uterine sarcoma (SDUS)	*SMARCA4* mutation	Chromatin remodeling/Cell cycle and growth pathways	EZH2 and CDK4/6 inhibitors
*COL1A1::PDGF* fusion uterine sarcoma	*COL1A1::PDGF* fusion	PDGF signaling and downstream PIK3/AKT pathway	TRK inhibitor
ERBB2/3 mutated S100/SOX10-positive uterine sarcoma	*ERBB2/3* mutations	PIK3/AKT/MAPK pathway	Her2 targeted therapy

Abbreviates: HRD: homologous recombination deficiency; MMR: mismatch repair.
